# Effects of adjacent land-use types on the distribution of soil organic carbon stocks in the montane area of central Taiwan

**DOI:** 10.1186/s40529-016-0147-5

**Published:** 2016-10-25

**Authors:** Chiou-Pin Chen, Kai-Wei Juang, Chih-Hsin Cheng, Chuang-Wen Pai

**Affiliations:** 1 Experimental Forest, College of Bio-resources and Agriculture, National Taiwan University, No. 12, Sec. 1, Chien-Shan Rd., Chu-Shan Township, Nan-Tou County, 55750 Taiwan; 2 Department of Agronomy, National Chiayi University, No. 300 Syuefu Rd., Chiayi City, 60004 Taiwan; 3 School of Forestry and Resource Conservation, National Taiwan University, No. 1, Sec. 4, Roosevelt Rd., Taipei, 10617 Taiwan

**Keywords:** Bamboo forest, Carbon to nitrogen ratio, Japanese cedar, Land-use type, Soil organic carbon stocks, Taiwania, Tea plantation

## Abstract

**Background:**

Soil organic carbon (SOC) stocks can be altered through reforestation and cropping. We estimated the effects of land use on SOC stocks after natural deciduous forests replaced by crops and coniferous plantations by examining the vertical distribution of SOC stocks at different depth intervals in an adjacent Oolong tea (*Camellia sinensis* L.) plantation, Moso bamboo (*Phyllostachys pubescens*) forest, Japanese cedar (*Cryptomeria japonica*) forest, and Taiwania (*Taiwania cryptomerioides*) forest in central Taiwan. The main soil characteristics, soil nitrogen (N) content, and soil carbon to nitrogen (C/N) ratio were also determined.

**Results:**

Different land uses resulted in significantly higher bulk density, lower cation exchange capacity, SOC, soil N, soil C/N ratio, and SOC stocks in croplands compared to forestlands. Due to the long-term application of chemical fertilizers, a significantly lower soil pH was found in the tea plantation. Croplands had a lower soil C/N ratio because of less C input into the soil and a higher mineralization rate of organic carbon during cultivation. Similar SOC stocks were found in Taiwania and Japanese cedar forests (148.5 and 151.8 Mg C ha^−1^, respectively), while the tea plantation had comparable SOC stocks to the bamboo forest (101.8 and 100.5 Mg C ha^−1^, respectively). Over 40% of SOC stocks was stored in croplands and over 56% was stored in forestland within the upper 10 cm of soil.

**Conclusions:**

Coniferous plantations can contribute to a higher SOC stock than croplands, and a significant difference can be found in the top 0–5 cm of soil.

**Electronic supplementary material:**

The online version of this article (doi:10.1186/s40529-016-0147-5) contains supplementary material, which is available to authorized users.

## Background

Carbon dioxide (CO_2_) can be released into the atmosphere via fossil fuel combustion and land-use changes to accelerate the greenhouse gas effect, leading to worldwide extreme weather, sea level rise, and habitation and ecological damage caused by global warming. In 2010, forests covered over 31% of land area in the world (FAO 2011) and thus have an important role in the C cycle. Since C is fixed as biomass C and soil organic carbon (SOC) in forest systems and by the end of the 20th century over 70% C was sequestrated in soil and peat deposits (Dixon et al. 1994), the role of forest SOC stocks and the possibilities of SOC loss caused by land use have been research areas of interest.

Land-use changes can accelerate SOC stock loss through erosion or vegetation conversion, and SOC stocks in surface soil and subsoil can change after native forest is converted to agricultural systems (Smith et al. 2002; Chen et al. 2004; Schulp et al. 2008; Fu et al. 2010; Don et al. 2011). Soil carbon content is known to decline once the natural forest has been cleared for croplands and plantations. Smith et al. (2002) suggested that converting large-scale of Amazonian tropical forest in Curuá-Una, Brazil to tree plantations of *Pinus caribaea* var. *hondurensis* Barrett and Golfari, *Euxylophora paraensis* Hub., *Carapa guianensis* Aubl., and Leguminosae decreased 9–13% C stocks in the 0–20 cm soils. Estimates of SOC stocks decline indicated by various researchers worldwide include an SOC stocks decrease of 13% from native forest to plantation and 42% from native forest to cropland (Guo and Gifford 2002), a decrease of 30% due to the conversion of forests to cultivated lands (Murty et al. 2002), and a 25 and 30% SOC loss for the conversion of primary forest to cropland and perennial crops, respectively (Don et al. 2011). In contrast, C can be restored in the mineral soil after cropland reverts to natural vegetation or is reforested to perennial vegetation (Post and Kwon 2000).

In Taiwan, only a few researches related to the effects of land-use change on SOC stocks have been published. Tsai et al. (2010) and Jien et al. (2010) estimated SOC stocks of 63 forest soils (excluding Histosols and Spodosols) and 140 cropland soils in the upper 100 cm depth, indicating that converting forestlands to croplands can significantly result in SOC stocks loss. Tsui et al. (2013) also estimated the SOC stocks under bamboo, grass, and secondary forests with the different elevation between 300 and 1000 m for Andisols and Inceptisols in Yangingshan National Park in northern Taiwan and suggested that elevation is a simple and effective predictor of SOC stocks. Lin et al. (2016) investigated SOC stocks in afforested and abandoned fields at different elevation and found that SOC stocks varied with elevation, land use, and soil depth.

Forests cover over 58% of the total land area of Taiwan (Taiwan Forestry Bureau 1994), and this amount of coverage would be expected to contribute to more SOC sequestration. However, crops and plantations have been widely replaced many of the native broad-leaved forests in the montane area in central Taiwan. Cash crops mostly tea tree and bamboo can be commonly found in the montane area at the elevation from 1000 to 1500 m because of their high economic value and the proper climate for their growth; therefore, many of local farmers use the forestlands illegally in an effort to increase economic benefits. Consequently, crops gradually replaced natural broad-leaved forests, and land overuse and abuse have become pervasive. Besides, most of the natural broad-leaved forests in the same area had been logged since the 1950s. To counter this, the Experimental Forest, National Taiwan University (NTU) has been reforesting land after the logging of natural forests and after reclaiming illegally used lands.

Since SOC stocks are likely to be different in these areas due to past tillage, long-term fertilization, afforestation, and reforestation (Guo and Gifford 2002; Smith et al. 2002; Chen et al. 2004; Degryze et al. 2004; Jandl et al. 2007; Jien et al. 2011) and because knowledge about SOC stocks of adjacent land-use types in the montane area in Taiwan is still scarce, obtaining information on the changes to SOC stocks in different land-use types is essential and imperative. The aim of this study was to investigate soil characteristics and the C/N ratio and to estimate the effects of land-use type on the distribution of SOC stocks in adjacent lands in the montane area of central Taiwan.

## Methods

### Study sites

**Fig. 1:**
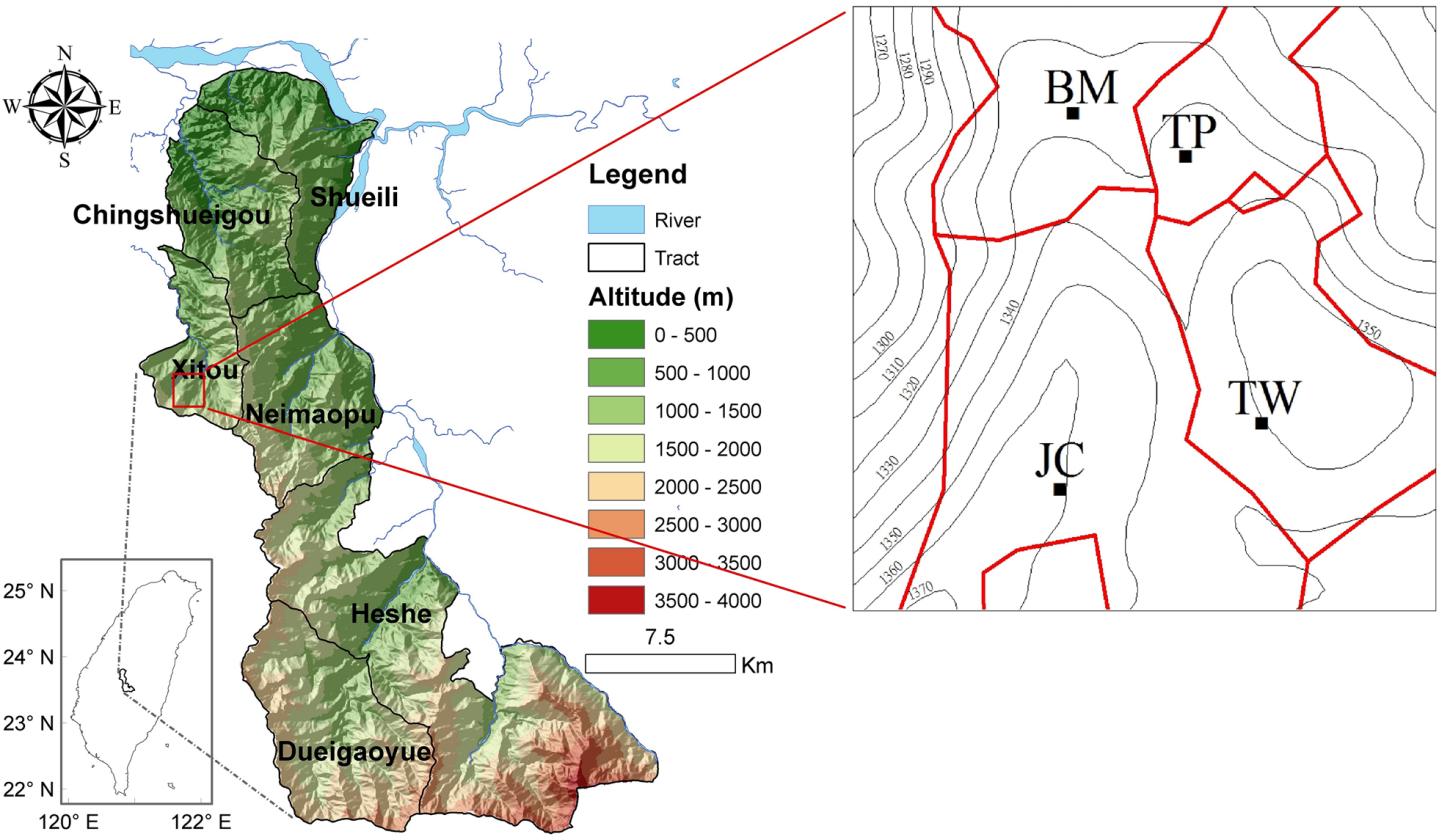
Location of the study area and sites, Xitou tract of Experimental Forest, National Taiwan University, Nantou County, Taiwan (*TP* tea plantation, *BM* Bamboo forest, *JC* Japanese Cedar, *TW* Taiwania)

This investigation was carried out in the Xitou tract of the Experimental Forest, NTU, Nantou County, central Taiwan (23°67′N, 102°77′E) (Fig. 1). The selected area has an elevation between 1200 and 1260 m above sea level and registers an average temperature of 16.6 °C and average annual precipitation of about 2600 mm. Soils are classified as Inceptisols, developed on sandstone, siltstone, and shale (Cheng et al. 2013).

To estimate the difference in SOC stocks among the local land uses of the selected area, we identified four land-use types as study sites: conventionally tilled cropland with Oolong tea (*Camellia sinensis* L.), conventionally tilled cropland with Moso bamboo (*Phyllostachys pubescens*), and two coniferous plantations with Japanese cedar (*Cryptomeria japonica*) and Taiwania (*Taiwania cryptomerioides*). Tea tree and bamboo are the predominant cash crops planted by local farmers because of their high economic value. Japanese cedar was first introduced from Japan in 1902, and Taiwania is a native pine species of Taiwan; both are the main coniferous species used for afforestation and reforestation by the Experimental Forest, NTU in Nantou.

Prior to the current land uses of our selected sites, native broad-leaved forests covered the area until being logged and converted to cropland and coniferous plantations. The selected coniferous plantations were reforested in 1973 and cropland sites have been tilled for over 40 years. Commercial fertilizers (Taiwan fertilizer company, No. 42. N–P_2_O_5_–K_2_O, N 22%, P_2_O_5_ 5%, K_2_O 6%) are applied four times a year to the tea plantation and once a year to the bamboo forest. The study sites were chosen because of their similar pedologic and climatic conditions for the different land-use types. The main environmental information of the four study sites is listed in Table 1.

**Table 1:** Environmental information of the four study sites

Land-use type	Altitude (m)	Slope (°)	Age (years)	Aspect	Dominant species and understory
Tea plantation	1200	5	40–50	NE	*Camellia sinensis* L.
Bamboo forest	1200	8	40–50	N	*Phyllostachys pubescens*
Japanese cedar forest	1260	6	42	SE	*Cryptomeria japonica* *Lasianthus fordii* Hance, *Elatostema lineolatum* Forst. *var. majus* Thwait.*, Diplazium virescens* Kunze, *Amischotolype hispida* (Less. & Rich.) Hong, *Schefflera octophylla* (Lour.) Harms, *Monachosorum henryi* Christ, *Lasianthus wallichii Wight, Zingiber kawagoii* Hayata
Taiwania forest	1240	10	42	SE	*Taiwania cryptomerioides* *Polygonum chinense* Linn., *Angiopteris lygodiifolia* Rosenst., *Diplazium dilatatum* Blume, *Ardisia cornudentata* Mez ssp. *morrisonensis* (Hayata) Yuen P. Yang var. *stenosepala* (Hayata) Yuen P. Yang, *Monachosorum henryi* Christ, *Elatostema lineolatum* Forst. var. *majus* Thwait., *Selaginella doederleinii* Hieron

### Soil sampling and analysis

In April 2015, three soil profiles were selected in a 25 × 20 m subplot of each site. Because slope can affect the estimation of SOC stocks, we chose profiles that had slopes between 5° and 10°. Soil carbon stocks within 0–50 cm soil of all land-use types was estimated for comparing soil carbon under the same depths since the depth of soil under bamboo forest was around 50 cm. Therefore, three samples were collected at specific intervals from the surface to 50 cm depth (0–5, 5–10, 10–30 and 30–50 cm) of each soil profile. Collected soil samples were air dried, ground, passed through a sieve (<2 mm), and stored for chemical and physical properties analyses and estimation of SOC stocks. Before soil sample collections, three samples at each depth were collected with volumetric cores for determining bulk density (BD). Core-collected soil samples were oven dried (105 °C) to constant weight prior to calculating their BDs (Blake and Hartge 1986). Particle size was determined by the pipette method (Gee and Bauder 1986). Soil pH was measured in deionized water (water–soil ratio 1:1) by glass electrode (McLean 1982). Soil samples were finely ground using a ball grinder (Oscillating Mill MM400; Retsch, Newtown, PA, USA) before SOC and N analysis. A CHN analyzer (Perkin Elmer 2400 CHN; Perkin Elmer, Norwalk, CT, USA) was used to analyze soil carbon and nitrogen. The CEC of the soil was carried out using NH_4_-acetate buffer solution (pH 7; Thomas 1982).

### SOC stock estimation

Soil organic carbon stocks within the 0–50 cm soil profiles of all land-use types were estimated for comparing SOC stocks at the same depths. Soil organic carbon stocks in the soil profiles were calculated as follows (Batjes 1996):

1 $$Td = \sum\limits_{i = 1}^{k} {\rho iPiDi\,\,\left( {1 - Si} \right)} ,$$

where *Td* = SOC stocks over depth (d) of layer (Mg m^−2^), *ρi* = BD of layer *i* (Mg m^−3^), *Pi* = C content of layer *i* (mg C g^−1^ soils), *Di* = thickness of layer *i* (m), and *Si* = percentage of rock fragment particles >2 mm in layer *i*.

### Statistical analysis

The effects of land use and soil depth on soil characteristics and SOC stocks in soil profiles were assessed by analysis of variance (ANOVA) using the generalized linear model (GLM) (SAS Institute Inc. 1985).

## Results

### Soil characteristics

The soil characteristics of the four study sites are listed in Additional file 1: Table S1). Soil texture was mostly sandy loam and loam, with sand being the dominant particle (60–84%) at all sites. All soil profiles showed similar trends of BD increasing with increasing depth and decreasing SOC. The BD of the croplands was generally higher than in the forestlands, and the difference was found to be significant in each layer (*p* < 0.001). Soil pH of the four study sites ranged from 2.8 to 4.2. A vertical gradient was observed for all soil profiles, with the lowest values in the surface soils and the highest in the deepest layers. Soil pH was negatively correlated with SOC in each profile. Japanese cedar and Taiwania forests had very similar soil pH at each depth. Among all study sites, the lowest and highest soil pH values were found at 0–5 cm in the tea plantation and at 30–50 cm in the bamboo forest (*p* < 0.001). In the four study sites, SOC ranged from 4.83 to 335 g kg^−1^, with the highest SOC found at 0–5 cm depth in all profiles and SOC decreasing with depth. The similar level of SOC in the forestlands was significantly higher than in the croplands (*p* < 0.001). Cation exchange capacity showed a positive correlation with SOC and a negative correlation with soil pH.

### Vertical distribution of soil N and C/N ratio

For all land-use types, soil N showed a similar distribution as SOC, with a decreasing trend from the surface layer to the deepest layer (Table 2). The croplands had a lower SOC levels and a lower soil N than the forestlands, with a significant difference found at 0–10 cm (0.52–1.03% for croplands and 0.8–2.82% for forestlands, respectively). With the exception of the Taiwania forest, the C/N ratios in the soil profiles of the different land-use types showed a decreasing tendency with depth. The soil in the Japanese cedar forest generally had the highest C/N ratios in each layer compared to the other land-use types. Croplands had lower soil C/N ratios than coniferous plantations; similar to the trend of N, the difference was distinct in the upper 0–10 cm.

**Table 2:** Soil N and C/N ratio at different soil depths in different land-use types

Land-use type	Depth (cm)	N (%)	C/N ratio
Tea plantation	0–5	1.03 (0.29)^a^	11.3 (0.8)
	5–10	0.52 (0.131)	10.7 (0.2)
	10–30	0.19 (0.025)	10.1 (0.4)
	30–50	0.11 (0.015)	8.20 (0.53)
Bamboo forest	0–5	0.77 (0.215)	10.6 (0.3)
	5–10	0.69 (0.057)	10.5 (0.5)
	10–30	0.26 (0.053)	9.93 (0.66)
	30–50	0.14 (0.025)	7.11 (0.79)
Japanese cedar forest	0–5	2.08 (0.54)	15.4 (0.3)
	5–10	0.80 (0.061)	13.1 (0.8)
	10–30	0.29 (0.064)	11.4 (1.4)
	30–50	0.16 (0.015)	8.65 (1.80)
Taiwania forest	0–5	2.82 (0.71)	12.2 (2.2)
	5–10	1.10 (0.39)	14.5 (1.9)
	10–30	0.26 (0.046)	10.9 (2.0)
	30–50	0.13 (0.031)	7.00 (2.12)
ANOVA for significance of			
Land-use type		JC = TW > TP = BM***	JC > TW > TP = BM***
Depth (cm)		0–5 > 5–10 > 10–30 = 30–50***	0–5 = 5–10 > 10–30 > 30–50***

*TP* tea plantation, *BM* bamboo forest, *JC* Japanese Cedar forest, *TW* Taiwania forest

*** p < 0.001

^a^Values in parentheses are stand errors (*n* = 3)

### Vertical distribution of SOC stocks

As shown in Table 3, the highest SOC stocks in the soil profiles of the four land-use types were mostly found in the 0–5 cm layers. Soil organic carbon stocks in the 0–5 cm layers of the croplands (21.2–33.6 Mg C ha^−1^) were significantly lower than those in the forestlands (55.3–58.4 Mg C ha^−1^). The trend of SOC stocks was generally in the order of Japanese cedar forest > Taiwania forest > tea plantation > bamboo forest for the 0–5-cm layer and Taiwania forest > Japanese cedar forest > bamboo forest > tea plantation for the 5–10 cm layer. Soil organic carbon stocks across the 0–50 cm depth for the four land-use types ranged from 100.5 to 151.8 Mg C ha^−1^, with the highest SOC stocks in the Taiwania forest, followed by the Japanese cedar forest and the tea plantation; the lowest stocks were found in the bamboo forest. The Taiwania forest and Japanese cedar forest stored the highest SOC stocks (151.8 ± 19 vs. 148.5 ± 28 Mg C ha^−1^, respectively), while the bamboo forest and the tea plantation stored the least (101.8 ± 15 vs. 100.5 ± 17 Mg C ha^−1^, respectively).

**Table 3:** SOC stocks (Mg C ha^−1^) in different land-use types

	Tea plantation (Mg C ha^−1^)	Bamboo forest (Mg C ha^−1^)	Japanese cedar forest (Mg C ha^−1^)	Taiwania forest (Mg C ha^−1^)
Depth (cm)				
0–5	33.6 (9.7)^a^	21.2 (5.70)	58.4 (15)	55.3 (11.5)
5–10	18.3 (3.0)	20.9 (3.74)	25.1 (3.0)	42.1 (14.3)
10–30	29.3 (6.6)	37.4 (8.96)	43.4 (8.0)	40.1 (9.1)
30–50	20.6 (4.4)	21.0 (5.6)	21.6 (6.9)	14.3 (2.6)
Total (0–50)	101.8 (15)	100.5 (17)	148.5 (28)	151.8 (19)
ANOVA for significance of				
Land-use type		JC = TW > TP = BM***		
Depth (cm)		0–5 = 10–30 > 5–10 > 30–50***		

*TP* tea plantation, *BM* Bamboo forest, *JC* Japanese Cedar forest, *TW* Taiwania forest

*** p < 0.001

^a^Values in the parentheses are stand errors (*n* = 3)

## Discussion

### Effects of land use on soil characteristics

As shown in Additional file 1: Table S1, BD and soil pH increased with soil depth while SOC and CEC decreased with soil depth in all land-use types. The significantly higher BD found in all soil layers in the croplands compared to the forestlands can be attributed to cultivation activities and less SOC content. Bulk density is directly affected by SOC content and cultivation and increased after the original forests were converted to cultivated land (Lal and Kimble 2001; Murty et al. 2002; Don et al. 2011). More SOC and less soil disturbance in the forestlands likely led to the lower BD at the same depth for these sites compared to the croplands. The lowest pH was found in the surface soil of the tea plantation. The significantly low soil pH at each depth in the tea plantation may be due to the long-term application of a chemical fertilizer since the intensive application of nitrogen fertilizers has been shown to decrease soil pH after an undisturbed forest was converted to a tea plantation (*Camellia sinensis*) (Bahrami et al. 2010). Low soil pH caused by the long-term application of a chemical fertilizer is commonly found in croplands throughout Taiwan. Bamboo forests received less fertilization and soil disturbance and thus were less affected by cultivation practices compared to the tea plantation.

Soil organic carbon showed a decreasing trend with increasing soil depth, in agreement with earlier studies (Yimer et al. 2007; Fu et al. 2010; Guan et al. 2015). The distinctly higher SOC content in the forestlands could be due to the higher C input and more soil organic matter accumulation compared to the croplands. On the other hand, the significantly lower SOC content in the croplands could be due to less C input, more soil disturbance, and a higher C mineralization rate caused by cultivation (Yimer et al. 2007; Sainju et al. 2008; Mancinelli et al. 2010). The negative correlation between soil pH and SOC implied that H^+^ released from soil organic matter could reduce pH since organic matter is one of the main sources of H^+^ in soil (Satrio et al. 2009). Nevertheless, compared with SOC, fertilizers were likely to have a greater effect on soil pH, particularly as the SOC content in the tea plantation was not as high as that in the Japanese cedar and Taiwania forests. The positive correlation between CEC and SOC and the negative correlation between CEC and soil pH was also found in all land-use types, in agreement with the results of Martel et al. (1978) and Bahrami et al. (2010). The low soil pH in the tea plantation was expected to contribute to a higher CEC, but this was not the case. Hence, we suggest that the SOC content had a stronger effect on the CEC than on soil pH in this study, and the higher CEC in coniferous plantations can be explained by their higher SOC content.

The results indicate that cultivation of croplands could lead to a lower CEC, less SOC, and a higher BD compared to coniferous plantations and a significant lower soil pH in tea plantation; these findings are in agreement with those of Lugo et al. (1986) and van Straaten et al. (2015) for estimating the effects of land-use change in subtropical soils and tropical forests.

### Effects of land use on soil N and C/N ratio

The declining trend in soil N, which was similar to the distribution of SOC in soil profiles, in all land-use types is in agreement with previous studies (Table 2) (Wang et al. 2012; Guan et al. 2015). Soil N and the soil C/N ratio in the croplands were significantly lower than those parameters in forestlands (*p* < 0.001). The marked effects of land use on soil N and C/N ratio in the upper 10 cm of soils indicated a greater C loss when the surface was disturbed from cultivation activities, such as plowing and harvesting in croplands. Tea leaves and bamboo shoots are usually harvested four times and twice a year, respectively, and a large amount of aboveground biomass is removed during harvesting and weeding. Our results are consistent with those of Saikh et al. (1998) who reported that deforestation and cultivation resulted in lower N and C/N ratio. The lower C/N ratio in croplands could be a consequence of less C input to the soil and a higher mineralization rate of organic carbon caused by more oxygen being introduced and a higher soil temperature during cultivation (Yimer et al. 2007). In contrast, the higher soil C/N ratio in forestlands was due to the distinct SOC accumulation and lower SOC decomposition rate. The C/N ratio can directly affect the SOC decomposition rate by changing the decomposer organisms, and the decomposition rate of SOC pool decreases greatly once the C/N ratio increases (Enríquez et al. 1993; Xu et al. 2016). Our results indicate that cultivation resulted in a lower soil N and C/N ratio in the croplands and that the higher C input and lower SOC decomposition rate in the forestlands led to the higher soil N and C/N ratio.

### Effects of land use on SOC stocks

We showed that coniferous plantations tended to accumulate more SOC and contribute to higher SOC stocks through the entire 0–50 cm soil profile (Table 3). The approximately 126 Mg C ha^−1^ of SOC stocks at 0–30 cm depth for Japanese cedar was in accordance with a previous study indicating SOC stocks for a 51-year-old Japanese cedar (*Cryptomeria japonica*) plantation ranged from 97.2 to 166 Mg C ha^−1^ in subtropical zone of northeastern Taiwan (Chang et al. 2006). Soil organic carbon stocks within the upper 50 cm of soils in forestlands were also significantly higher than in croplands, although tea plantation and bamboo forest had a higher BD, which contributed to higher SOC stocks. Guan et al. (2015) also estimated SOC stocks in coniferous and bamboo plantations in mid-subtropical area, southeastern China and found that a Chinese fir (*Cunninghamia lanceolata* [Lamb.] Hook) plantation had higher SOC stocks than a Moso bamboo (*Phyllostachys heterocycla* [Carr] Mitford *cv*. *Pubescens*) plantation after natural forest conversion. However, the difference was not significant since the time span after forest conversion was 17 and 18 years, respectively. The longer land-use history (over 40 years) of our study sites could have led to the greater difference between the coniferous plantations and the bamboo forest. The variation of SOC stocks at each depth in the four land-use types, especially in the upper 5 cm, indicated that land use affected the SOC stocks and that forestlands accumulated much more SOC than croplands in the surface soils.

The removal of the understory vegetation in the croplands from cultivation and weeding and the harvesting of tea and bamboo each year likely removed a significant amount of biomass and reduced C and litter input to the soil (Guan et al. 2015). Aside from cultivation and harvesting, allelopathic compounds released from bamboo during leaf decomposition could also reduce C input. Allelopathic compounds released from bamboo (*Phyllostachys edulis* (Carr.)) leaves can reduce the seedling abundance and species richness and then to prevent the growth of understory plants, thus reducing litter; this would also reduce the input of SOC in the bamboo forest (Chou and Yang 1982; Chang and Chiu 2015). Meanwhile, the diversity and composition of soil bacterial communities could be different in coniferous plantations and croplands, which could affect the SOC decomposition rate and then the SOC stocks. Lin et al. (2014a) indicated that Japanese cedar (*Cryptomeria japonica*) and Moso bamboo (*Phyllostachys eduis*) forests in central Taiwan showed the different diversity and composition of soil bacterial communities because of the intensive management to increase soil disturbances in the bamboo forest. Lin et al. (2014b) also suggested that the diversities of bacterial communities of the replanted *Calocedrus formosana* and *Cryptomeria japonica* forests increased compared to natural hardwood forest in a perhumid area in northern Taiwan.

Furthermore, intensive agricultural practices in tea plantation and bamboo forest could also lead to lower SOC. Intensive practices, such as annual applications of inorganic fertilizers and deep tillage, would improve aeration in the soil profile and thus increase the rate of decomposition of soil organic matter, reducing SOC stocks (Sainju et al. 2008; Mancinelli et al. 2010). Chang and Chiu (2015) indicated that frequent human disturbances such as bamboo shoot harvesting could accelerate the degradation of soil organic matter in a Moso bamboo (*Phyllostachy edulis*) forest in central Taiwan. Li et al. (2013) also indicated that long-term intensive practices reduced SOC stocks in Moso bamboo (*Phyllostachys pubescens*) forests in southeastern China. Removal of understory vegetation in croplands not only reduced C input but also increased soil temperature, which enhanced mineralization of soil organic matter and led to a decrease in SOC and SOC stocks (Wang et al. 2011). In contrast, less soil disturbance and a slower SOC decomposition rate in forestlands may result in a greater accumulation of SOC and thus contribute to greater SOC stocks. The finding of different SOC stocks in croplands compared to forestlands is consistent with the results of previous studies. After natural forests were converted to different land-use types, SOC stocks in croplands decreased more than in plantations (Guo and Gifford 2002; Don et al. 2011). Thus, cultivation in this area by local farmers resulted in a decrease in SOC stocks, whereas coniferous reforestation can store more SOC and somehow alleviate SOC losses.

## Conclusions

Long-term cultivation in croplands significantly led to lower soil pH, CEC, SOC, soil N, soil C/N ratio, and SOC stocks but higher BD compared to coniferous plantations. We suggest that the variation in SOC stocks in different land-use types is mainly affected by less soil disturbance and higher SOC content in the forestlands and less C input and higher C mineralization in the croplands because of less understory vegetation, more harvesting, allelopathy, and intensive agricultural practices. Taiwania forest stored the highest SOC stocks similar to Japanese cedar forests, while the bamboo forest and tea plantation had the lowest SOC stocks. Soil organic carbon stocks at 0–50 cm depth in coniferous plantations were significantly higher than in croplands, with a significant difference found in the upper 5 cm. Our data indicate that cropping will result in less SOC stocks compared to reforestation and that Taiwania and Japanese cedar forests have the potential to sequester more SOC than tea plantation and bamboo forest, particularly in the montane area of central Taiwan.

## Additional file

**Additional file 1: Table S1.** General physical and chemical properties of soil pedons in different land-use types.
