# Supplementary material for: Effects of adjacent land-use types on the distribution of soil organic carbon stocks in the montane area of central Taiwan
Source: Bot Stud. 2016 Oct 25;57:32. doi: 10.1186/s40529-016-0147-5 (PMC5430586; doi:10.1186/s40529-016-0147-5)
Supplement: Supplementary file 1 — Additional file 1: Table S1. General physical and chemical properties of soil pedons in different land-use types. [file 40529_2016_147_MOESM1_ESM.doc]

Table S1. General physical and chemical properties of soil pedons in different land-use types

| Land-use  type | Depth  (cm) | | Particle size analysis | | | Texture | BD^c^ | pH | SOC | CEC^d^ |
| --- | --- | --- | --- | --- | --- | --- | --- | --- | --- | --- |
|  |  |  | Sand Silt Clay | | |  |  |  |  |  |
|  |  | | -----------%---------- | | |  | (Mg m^-3^) |  | (g kg^-1^) | (cmol ^(+)^ kg^-1^) |
| TP^a^ | 0–5 | | 68 | 18 | 14 | SL^b^ | 0.58 (0.04)^e^ | 2.8 (0.2) | 117 (37) | 43.8 (8.0) |
|  | 5–10 | | 62 | 22 | 16 | SL | 0.69 (0.07) | 2.9 (0.2) | 55.2 (13.2) | 32.6 (6.4) |
|  | 10–30 | | 64 | 20 | 16 | SL | 0.80 (0.08) | 3.2 (0.2) | 19.4 (2.2) | 20.7 (3.4) |
|  | 30–50 | | 62 | 20 | 18 | SL | 1.1 (0.0) | 3.6 (0.1) | 9.33 (1.80) | 13.3 (0.2) |
| BM | 0–5 | | 66 | 24 | 10 | SL | 0.53 (0.07) | 3.5 (0.1) | 81.8 (23.8) | 30.6 (2.7) |
|  | 5–10 | | 62 | 22 | 16 | L | 0.60 (0.09) | 3.5 (0.0) | 72.8 (6.8) | 27.9 (1.6) |
|  | 10–30 | | 64 | 20 | 16 | SL | 0.80 (0.05) | 4.0 (0.1) | 25.9 (6.2) | 18.9 (4.6) |
|  | 30–50 | | 66 | 18 | 16 | SL | 1.1 (0.1) | 4.2 (0.2) | 9.80 (2.70) | 12.9 (0.4) |
| JC | 0–5 | | 84 | 14 | 2 | LS | 0.37 (0.08) | 3.5 (0.2) | 321 (87) | 70.7 (5.5) |
|  | 5–10 | | 60 | 24 | 16 | L | 0.52 (0.07) | 3.3 (0.0) | 104 (5) | 35.6 (3.8) |
|  | 10–30 | | 70 | 14 | 16 | SL | 0.73 (0.08) | 3.8 (0.1) | 33.9 (10.6) | 22.5 (1.6) |
|  | 30–50 | | 66 | 16 | 18 | L | 0.91 (0.10) | 4.0 (0.1) | 14.3 (4.4) | 15.8 (3.1) |
| TW | 0–5 | | 78 | 16 | 6 | SL | 0.34 (0.10) | 3.2 (0.1) | 335 (31) | 79.8 (6.4) |
|  | 5–10 | | 62 | 20 | 18 | L | 0.60 (0.05) | 3.1 (0.1) | 164 (80) | 55.6 (15.1) |
|  | 10–30 | | 64 | 20 | 16 | L | 0.75 (0.06) | 3.9 (0.2) | 28.0 (7.6) | 21.5 (3.6) |
|  | 30–50 | | 62 | 20 | 18 | L | 0.88 (0.09) | 3.9 (0.2) | 8.46 (0.70) | 13.4 (2.6) |
| ANOVA for significance of: | | | | | | |  | | | |
| Land-use type | |  | |  | | | (TP = BM > JC = TW)*** | (TP < JC = TW < BM)*** | (JC = TW > TP = BM)*** | (TW > JC > TP > BM)*** |
| Depth (cm) | |  | |  | | | (0–5 < 5–10 < 10–30 < 30–50)*** | (0–5 = 5–10 < 10–30 < 30–50)*** | (0–5 > 5–10 > 10–30 = 30–50)*** | (0–5 > 5–10 > 10–30 > 30–50)*** |

^a^TP=Tea plantation, BM=Bamboo forest, JC=Japanese Cedar forest, TW=Taiwania forest; ^b^ SL=Sandy Loam, LS=Loamy Sand, L=Loam; ^c^BD=Bulk Density; ^d^CEC=Cation Exchange Capacity

^e^Values in parentheses are standard errors (*n*=3)

****p*<0.001
